# The association between kidney function, cognitive function, and structural brain abnormalities in community-dwelling individuals aged 50+ is mediated by age and biomarkers of cardiovascular disease

**DOI:** 10.1093/cvr/cvad060

**Published:** 2023-04-13

**Authors:** Natalia Nowak, Celine De Looze, Aisling O’Halloran, Rose Anne Kenny, Donal J Sexton

**Affiliations:** The Irish Longitudinal Study on Ageing, Trinity College Dublin, D02 R590, Dublin 2, Ireland; School of Medicine, Trinity College Dublin, College Green, D02 PN40, Dublin 2, Ireland; The Irish Longitudinal Study on Ageing, Trinity College Dublin, D02 R590, Dublin 2, Ireland; School of Medicine, Trinity College Dublin, College Green, D02 PN40, Dublin 2, Ireland; The Irish Longitudinal Study on Ageing, Trinity College Dublin, D02 R590, Dublin 2, Ireland; School of Medicine, Trinity College Dublin, College Green, D02 PN40, Dublin 2, Ireland; The Irish Longitudinal Study on Ageing, Trinity College Dublin, D02 R590, Dublin 2, Ireland; School of Medicine, Trinity College Dublin, College Green, D02 PN40, Dublin 2, Ireland; Saint James Hospital, Dublin, D08 NYH1, Dublin 8, Ireland; The Irish Longitudinal Study on Ageing, Trinity College Dublin, D02 R590, Dublin 2, Ireland; School of Medicine, Trinity College Dublin, College Green, D02 PN40, Dublin 2, Ireland; Saint James Hospital, Dublin, D08 NYH1, Dublin 8, Ireland

**Keywords:** Vascular risk factor, GDF15, Kidney function, Ageing, Cerebrovascular disease

## Abstract

**Aims:**

Cognitive impairment has been associated with kidney function and chronic kidney disease. Whether this association is due to accelerated cardiovascular disease (CVD) or an independent specific kidney function effect related to toxins is unclear. We investigated the impact of an array of clinical factors, inflammatory biomarkers, and cardiovascular biomarkers on the association between kidney function, cognitive function, and structural brain abnormalities.

**Methods and results:**

We used data from the first and third waves of the TILDA Study, a population-representative prospective cohort of Irish adults aged 50 years and over, based on stratified random sampling (*n* = 3774). The MRI sub-study included participants who consented to MRI brain imaging in addition to the health assessment. Multivariable linear and mixed-effect longitudinal regression models were fitted separately for each kidney marker/estimated glomerular filtration rate (eGFR) equation after adjusting for baseline age and demographics, clinical vascular risk factors, and biomarkers. Unadjusted analyses showed an association between low eGFR, cognitive dysfunction, and cognitive decline (*P* < 0.001 for all kidney markers). Kidney function markers were also associated with white matter disease [OR = 3.32 (95% CI: 1.11, 9.98)], total grey matter volume (β = -0.17, 95% CI -0.27 to -0.07), and regional grey matter volumes within areas particularly susceptible to hypoxia (*P* < 0.001 for all). All the associations decreased after adjusting for age and were also diminished after adjusting for CVD biomarkers. Age and CVD-biomarker score were significant mediators of the adjusted associations between eGFR and cognitive status. These results remained consistent for cross-sectional and longitudinal outcomes and specific cognitive domains.

**Conclusion:**

Decreased kidney function was associated with cerebrovascular disease. The association appeared to be mediated predominantly by age and the combination of CVD markers [namely N-terminal pro-B-type natriuretic peptide (NT-proBNP) and Growth Differentiation Factor 15 (GDF15)], supporting the idea that shared biological pathways underline both diseases. Further mechanistic studies of the specific molecular mechanisms that lead to both kidney and cognitive decline are warranted.

## Graphical Abstract

**Graphical Abstract:**
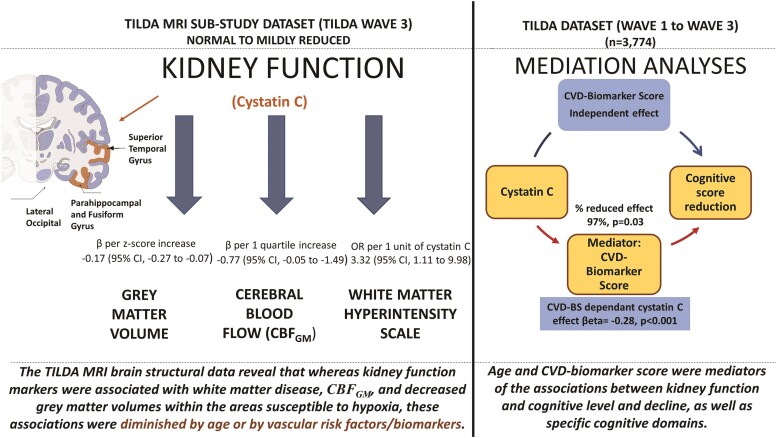

**Time of primary review: 37 days**

## 1. Introduction

Robust associations of severely impaired estimated glomerular filtration rate (eGFR) with cognitive disorders, dementia, and cerebrovascular events are well established.^1,2^ Less is known about the relationship between more modest reductions in kidney function and neuro-psychological degeneration, as well as the underlying mechanisms.

A proposed process that immediately precedes clinically significant kidney and cognitive impairment is subclinical cardiovascular disease (CVD), a process that affects blood flow in both organs.^3^ Individuals with chronic kidney disease (CKD) are at elevated risk of CVD at all disease stages however endothelial injury likely initiates while kidney function and cognitive function are relatively preserved. These individuals may then progress to develop clinically significant vascular complications at a later stage which might predominate in peripheral vascular, coronary vascular, reno-vascular, or cerebrovascular disease.^4,5^ In those individuals with concomitant kidney dysfunction and cognitive impairment, reduced eGFR could either be considered as an independent risk factor for the incidence of cognitive abnormalities or it may reflect an indirect measure of the severity of vascular ageing or comorbid atherosclerotic processes contributing to a more rapid cognitive decline.^3^

Previously, cystatin C-estimated GFR (eGFRcys) showed larger effect size in relation to cognitive function loss compared with creatinine eGFR (eGFRcre).^6–8^ How eGFR relates to specific vascular factors, in the general population, is uncertain. Such a relationship could explain, in part, the association of kidney function with increased odds of cognitive loss. Some data suggested that the relationship between eGFRcys and incident dementia remained significant after accounting for lipids, C-reactive protein (CRP), diabetes, and hypertension.^6–8^ Whether the association between kidney function and cognitive decline is mediated by ageing itself, inflammatory processes, vascular disease, or a combination thereof has not been investigated at a population level to date.

Given the ongoing debate as to whether a common biological pathway can be determined that underlies the relationship of kidney decline and cognitive decline, we aimed to explore how kidney dysfunction and brain dysfunction are linked in a large cohort of healthy people aged over 50 years who enrolled in the population-based Irish Longitudinal Study on Ageing (TILDA) between 2009 and 2011.^9,10^ First, we investigated the associations between different renal markers and cognitive level and decline, including global cognition, memory, and executive function. Secondly, we evaluated the independence of associations between kidney function and MRI brain structural abnormalities [i.e. regional grey matter volumes, white matter hyperintensities, and grey matter cerebral blood flow (CBF_GM_)]. Finally, we performed mediation analyses to assess the effect of an array of vascular factors (clinical vascular risk factors presumed from clinical characteristics, inflammatory biomarkers, and cardiovascular biomarkers [i.e. Growth Differentiation Factor 15 (GDF15), and N-terminal pro-B-type natriuretic peptide (NT-proBNP)] previously associated with cognitive decline) on the association between cystatin C, cognition, and cognitive decline.^11–13^ We were particularly interested in evaluating the importance of vascular risk factors to examine the hypothesis that the mechanisms involved in vascular disease might be involved in the initiation of cognitive decline in individuals with mildly reduced renal function in a community-dwelling cohort.

## 2. Methods

### 2.1 Study population

TILDA is a cluster-sampled national prospective cohort study of community-dwelling adults aged 50 years and over based on stratified random sampling. Households were selected in geographic clusters from a list of all residential addresses in Ireland. Detailed descriptions of the recruitment protocol have been published previously.^14^ Briefly, between October 2009 and February 2011, 8175 participants completed a home-based, computer-assisted personal interview and were subsequently invited to take part in a comprehensive health assessment (HA) in a dedicated health center, carried out by trained nurses.^14^ Each respondent provided written informed consent. Those with cognitive impairments that prevented meaningful consent from being given were not included in the study. Our analysis of cognitive outcomes included participants who completed HA and had kidney function available at both waves. The MRI sub-study included participants who consented to MRI in addition to HA. The participants provided informed signed consent. The Research Ethics Committee of Trinity College Dublin approved the study protocol. All experimental procedures adhered to the Declaration of Helsinki.

### 2.2 Kidney function

Plasma creatinine and cystatin C were measured with an enzymatic and a particle-enhanced immunonephelometric assay, respectively. The measurements were performed in the same blood sample, at the Clinical Laboratory, at Saint James Hospital, Dublin. The creatinine measurement method aligned with calibration to IDMS (Roche Creatinine plus ver.2, Roche Diagnostics, Basel, Switzerland). We estimated eGFRcre with the Chronic Kidney Disease Epidemiology Collaboration (CKD-EPI) formula,^15^ 4-variable MDRD formula,^16^ and Berlin Initiative Study formula^17^; and from cystatin C with CKD-EPI formula (CKD EPI-eGFRcys).^18^ Additionally, eGFR on the basis of the CKD-EPI equation was calculated for both measurements combined (CKD-EPI eGFRcrcys)^16^; CKD was defined as CKD-EPI eGFRcrcys < 60 mL/min.

### 2.3 Covariates/mediators

#### 2.3.1 Clinical characteristics

Participant clinical characteristics included age, sex, educational attainment, self-reported depression measured with a Center for Epidemiologic Studies Depression scale,^19^ smoking, and self-reported physician-diagnosed conditions. For the definition of abnormal body mass index (BMI), we used a cut-off of 25 kg/m^2^. Seated systolic and diastolic blood pressure, and pulse wave velocity between the carotid and femoral arteries (cf-PWV) were measured at HA.^14^ Cf-PWV was measured by tonometry (Vicorder) and the average of two measurements between the carotid and femoral arteries was used. Serum total and the high-density lipoprotein (HDL) cholesterol were measured from each sample before freezing with an automated enzymatic method.

#### 2.3.2 Cardiovascular and inflammatory biomarkers

Hypotheses relating to the pathophysiology of cognitive decline associated with reductions in kidney function tend to focus largely on three broad and overlapping domains, that of CVD, systemic inflammation, and ageing.^2^ Therefore, we choose to explore the effect of biomarkers of inflammation and CVD on this association, in addition to age. The marker concentrations were measured in plasma specimens obtained at study enrollment and stored in -80°C until the measurements were performed. The determinations were performed with Meso Scale Discovery ELISA-based assay multiplex platform, at Cardiff Laboratory. The coefficients of inter-assay variability for all the biomarkers are presented in *Supplementary Methods* Section. The biomarkers were standardized to a mean of 0 and a standard deviation of 1 to enable a more meaningful comparison of their effect sizes. The biomarker scores were calculated by summing standardized scores of individual process-specific biomarkers and included: (i) Tumor Necrosis Factor-alpha, Interleukin-6, Interleukin-8, and Interleukin-1 Receptor Antagonist for inflammatory biomarker score (**Inflammation BS**); and (ii) NT-proBNP and GDF15 for cardiovascular biomarker score (**Cardiovascular BS**).

### 2.4 Study outcome

#### 2.4.1 Cognitive outcomes

Cognitive function was assessed with the following neuropsychologic test battery: Montreal Cognitive Assessment (MoCA), Mini-Mental State Exam (MMSE), Immediate word recall task (comprising the sum of two recall trials), Delayed word recall task, Colour Trial 1 Test, Colour trials 2 test, and Sustained Attention to Response Task (SART) Test.^18^ The composite score for **memory** was the average of the *z*-scores for the Immediate and Delayed word recall task tests. The composite score for **executive function/attention** was constructed by averaging the *z*-scores for the time to complete Colour Trial 1 Test, Colour Trial 2 test, and mean response time on SART test. The **global-function** composite score was the average of the z-sores from MoCA and MMSE.^20^

#### 2.4.2 Brain imaging

We extracted the data on brain structural outcomes: CBF_GM_, global white matter hyperintensity (WMH), as well as grey matter volumes (for the entire cortex and the function-specific regions) from the TILDA MRI sub-study dataset. The cohort in this MRI sub-study comprised 560 Wave 3 TILDA (2014–2015) participants. Briefly, of 4309 participants attending for HA, a random subset was invited to return for a multi-parametric brain MRI at the National Centre for Advanced Medical Imaging, Dublin, Ireland. The detailed protocol of MRI scanning was described in previous publications and included a variety of scans: FLAIR, pCASL (pseudo-continuous ASL), and a T1-weighted MR image acquired using a 3D Magnetisation Prepared Rapid Gradient Echo sequence, with the following parameters: FOV (mm): 240 × 218 × 162; 0.9-mm isotropic resolution; SENSE factor: 2; TR: 6.7 ms; TE: 3.1 ms; flip angle: 9.^21–23^ All MRI examinations were performed on a 3T system using a 32-channel head coil. Mean whole-brain CBF_GM_ was represented in voxel-wise absolute perfusion values in mL/100 g/min.^22^ WMH was assessed visually from the MRI images, by trained radiologists, and was expressed via Scheltens’ semi-quantitative rating scale^24^; the WMH data were available for 380 participants with kidney markers determinations. Grey matter volumes were measured automatically with FreeSurfer pipeline^25,26^; cortical and subcortical segmentations (Desikan Atlas within^27^) were manually inspected using Freeview. We further normalized raw regional and total grey matter volumes by dividing the grey matter volume by the estimated intracranial volume, to correct for the differences associated with head size. Total regional grey matter volumes were generated using the sum of their left and right hemisphere respective volumes.

### 2.5 Statistical approach

The statistical analyses were performed in SAS Version 9.4 (SAS Institute, Cary, NC). Clinical characteristics were reported as counts and percentages (proportions) for categoric variables, and medians (inter-quartile range) for continuous variables. Comparisons between medians were done with the Wilcoxon test, categorical variables were compared with the χ2 test. Differences in individual cognitive test scores between quartiles of cystatin C were tested with the Jonckheere–Terpstra trend test.

#### 2.5.1 Analysis of cognitive outcomes

For the analyses of the decline in global cognitive scores, participants were followed from the baseline visit (Wave 1) until Wave 3 (2014–2015). Multivariable regression analysis was used to model neurocognitive study outcomes, with continuous variables standardized by transformation to *z*-scores. For longitudinal measures of transformed MoCA/MMSE (global cognition), we fitted a linear mixed-effects model (PROC MIXED, SAS 9.4, SAS Institute, Cary, NC). We modelled kidney function expressed as crude cystatin C concentration, CKD-EPI eGFRcys formula, CKD EPI eGFRcre formula, MDRD formula, and BIS eGFRcre formula. We utilized a nested adjustment scheme to evaluate the potential confounding characteristics. The set of variables/confounders was chosen based on data availability and previously existing evidence of their association with cognitive decline. Model 1 was adjusted for age and demographics (i.e. sex, depression, educational attainment), and baseline cognitive score. Model 2 was adjusted for the variables in model 1 and clinical vascular risk factors (i.e. smoking, diabetes, blood pressure, obesity, number of CVD risk factors, cholesterol, HDL cholesterol, and CRP). Model 3 was adjusted for the variables in Models 1 and 2, and biomarkers (i.e. Inflammation BS and Cardiovascular BS). There were few missing variables for the analysis. Pulse wave velocity, which was measured in the health centre, was missing in 12% of the entire cohort used in the analyses of cognitive function/cognitive decline; missingness was shifted towards participants with impaired kidney function, and as such, the variable was excluded from the main data presentation. Multicollinearity was quantified with variance inflation factors (VIF). The cognitive domains (i.e. *z*-scores for global, memory, and executive function performance) were modelled using linear regression and nested adjustment model described above. Sensitivity analyses were performed for patients without any diagnosed/reported CVD conditions [i.e. angina, stroke, TIA, heart failure, or heart attack (including myocardial infarction or coronary thrombosis)].

#### 2.5.2 Analysis of brain structural data

We then assessed the confounding variables of the association of kidney function with brain outcomes in distinct areas as assessed by MRI in a subset of the entire cohort. We performed univariable, and multivariable analyses for regional grey matter volumes, white matter structure, and cerebral blood flow. Multivariable regression models tested associations of each kidney marker (cystatin C, CKD-EPI eGFRcys, or CKD-EPI eGFRcre) with the structural brain outcomes acquired at TILDA Wave 3. Two etiological models were built and were separately adjusted for (i) age and demographics (i.e. sex, education, and depression scale), or (ii) for clinical vascular factors (i.e. smoking, diabetes, blood pressure, obesity, number of CVD risk factors, cholesterol, HDL cholesterol, pulse wave velocity, CRP concentration), and biomarkers (i.e. Inflammation BS, and Cardiovascular BS). WMH was a semi-quantitative variable, expressed via Scheltens’ visual rating scale. Owing to the nature of this scale (i.e. non-equal spacing of unit-wise increments) and skew in the data, Schelten’s score variable was converted into an ordinal dependent measure (Score 0–9, 10–19, 20–29, and ≥30 mm); this ordinal measure was then modelled with ordered logistic regression. Associations with quantitative outcomes (i.e. grey matter volumes and CBF_GM_) were tested with linear regression and expressed as standardized coefficients (βeta-estimates) per increase of *z*-score transformed grey matter volumes and each kidney marker, with corresponding 95% CIs (one degree of freedom). In the analysis of grey matter volumes within different functional regions, we accounted for potential false positives due to multiple comparisons by utilizing Bonferroni-adjusted *P*-values (adjustment yielded a threshold of <1.5 × 10^−3^). We performed a series of sensitivity analyses excluding subjects with self-reported history of stroke and Parkinson’s disease (*n* = 3).

#### 2.5.3 Mediation analysis

Mediation analysis informs about the potential mechanisms by which a given exposure influences an outcome and may support screening whether intervention on certain mediator could possibly alleviate the effect of the exposure.^28^

We performed a series of mediation analyses to estimate what fraction of the association between cystatin C and cognitive performance can be explained by each of the vascular factors as mediators.^29,30^ We built linear regression models with cognitive outcomes as a dependent variable. Age, systolic blood pressure, diastolic blood pressure, CRP, Inflammation BS, and Cardiovascular BS were tested as mediators of cystatin C effects. In theory, a simple mediation analysis relies on the two regression equations: *E*(*M*|*A* = *a*, *C* = *c*) = β0 + β1*a* + β′2*c* and *E*(*Y*|*A* = *a*, *M* = *m*, *C* = *c*) = θ0 + θ1*a* + θ2*m* + θ′4*c* (where *M* is the mediator, *A* the exposure, *C* the confounders, *Y* the outcome, and the vertical line represents the model conditional upon the following variable) to estimate the total effect of given exposure and its decomposed effects [i.e. natural direct effect (NDE), and natural indirect effect (NIE)]. The NDE is expressed as NDE = θ1(*a*–*a*∗), and the NIE as NIE = θ2β1(*a* - *a*∗).^28^ When considering causal interpretation, one needs to assume that the regression models were correctly specified, that the analysis was controlled for confounding, and that mediator-outcome confounders were not influenced by the exposure. The cohort of 3774 individuals was used in our mediation analyses. The models were adjusted for relevant covariates (the covariates specified in the Multivariate Models 1 and Model 2 in Paragraph 2.5.1). The natural log-transformed value of cystatin C (ln-cystatin C) at TILDA Wave 1 was tested as an exposure, the specific cognitive outcome variables included global cognition, memory, executive function, and the difference in global cognition, memory, and executive function between TILDA Wave 1 and TILDA Wave 3. The total and decomposed effects of kidney function were expressed per a one mg/mL unit increase of ln-cystatin C. Additional sensitivity analyses were performed for patients without any diagnosed/reported CVD conditions. We had low statistical power to perform mediation using MRI data as an outcome.

## 3. Results

Of the 3774 participants included in the analysis of cognitive outcomes, mean age was 62^8^ years, mean eGFRcyscre was 82^14^ mL/min, 6.2% had diabetes, and 34% meet the criteria for hypertension. Median cystatin C and cystatin C eGFR were 0.92 mg/L and 82.7 mL/min, respectively. A total of 9.5% participants have met CKD criteria. *Table 1* shows a summary of clinical variables for the study cohort, stratified according to quartiles of cystatin C. Unadjusted testing results showed significant differences among the cystatin C groups for the parameters in *Table 1*. Most factors known to be associated with vascular risk tended to be increased in the higher cystatin C strata: age, BMI, systolic blood pressure, CRP, pulse wave velocity, cardiovascular BS, and inflammation BS, whereas CKD-EPI eGFRcys, all eGFRcre formulas, and lipids, were found to be lower. People at higher cystatin C strata tended to be diabetic and hypertensive. Additionally, the individual item cognitive scores (individual tests for global cognition, memory, and executive function) were gradually decreasing with increasing cystatin C strata (see Supplementary material online, *Table S1*).

**Table 1:** Clinical characteristics and cardiovascular risk factors in the study population at wave 1

Cystatin C at Wave 1	1st quartile	2nd quartile	3rd quartile	4th quartile
No of individuals	970	953	964	887
Age (yr)	57 (53, 62)	59 (54, 64)	62 (57, 68)	68 (62, 75)
Sex (M/F)	359/611	444/509	506/458	448/439
Cystatin C (mg/L)	0.79 (0.74, 0.82)	0.89 (0.87, 0.91)	0.99 (0.96, 1.02)	1.17 (1.10, 1.30)
EPI-eGFRcys (mL/min)	102.5 (98.4, 107.1)	88.6 (84.6, 91.9)	76.7 (73.1, 80.1)	59.9 (51.5, 63.3)
EPI-eGFRcre (mL/min)	93.4 (85.1, 99.2)	86.9 (77.3, 94.5)	79.1 (71.7, 87.2)	66.2 (55.1, 76.1)
BIS-eGFRcre (mL/min)	89.2 (80.7, 99.2)	81.2 (73.1, 90.9)	74.1 (67.0, 82.1)	60.6 (52.7, 70.4)
MDRD eGFR (mL/min)	87.2 (78.5, 96.8)	81.0 (72.0, 90.0)	74.4 (67.9, 82.6)	63.9(54.5, 72.6)
Education (n/%)				
primary	152 (16)	177 (18)	221 (23)	288 (32)
secondary	397 (41)	387 (42)	371 (38)	344 (39)
higher	420 (43)	389 (40)	372 (39)	255 (29)
Smoking (yes %)	47%	55%	54%	60%
Diabetes (yes %)	4.5	5.9	4.7	10.5
BMI (kg/m^2^)	26.5 (24.3, 29.6)	27.7 (25.0, 30.3)	28.2 (25.6, 31.1)	29.3 (26.5, 32.8)
BMI > 25 (%)	68	75	79	85
Systolic BP (mmHg)	130 (118, 143)	132 (120, 146)	134 (122, 148)	138 (125, 152)
Diastolic BP (mmHg)	81 (74, 88)	82 (75, 89)	82 (75, 90)	82 (74, 90)
Cholesterol (mmol/L)	5.3 (4.7, 5.9)	5.2 (4.4, 5.9)	5.2 (4.4, 5.9)	4.8 (4.1, 5.7)
HDL-C (mmol/L)	1.6 (1.4, 2.0)	1.5 (1.2, 1.7)	1.5 (1.2, 1.8)	1.4 (1.1, 1.7)
LDL-C (mmol/L)	3 (2.4, 3.6)	3 (2.4, 3.6)	2.9 (2.3, 3.6)	2.7 (2.1, 3.3)
C-reactive protein (µg/mL)	1.1 (1.0, 2.2)	1.4 (1.0, 2.9)	1.5 (1.2, 1.8)	2.4 (1.2, 4.6)
Pulse wave velocity (m/s)	9.5 (8.5, 10.8)	10.0 (8.8, 11.3)	10.3 (9.1, 11.7	11.1 (9.9, 12.6)
CVD biomarker score	-0.72 (-0.95, -0.36)	-0.60 (-0.83, -1.16)	-0.37 (-0.71, 0.16)	0.35 (-0.24, 1.54)
Inflammation biomarker score	-0.65 (-0.93, -0.10)	-0.54 (-0.83, -0.05)	-0.46 (-0.79, 0.07)	-0.06(-0.53, 0.54)

**Abbreviations:** BP; blood pressure, BMI; body mass index, CVD; Cardiovascular Disease, cys; cystatin C, cre; creatinine, EPI-eGFR; Chronic Kidney Disease Epidemiology Collaboration- estimated Glomerular Filtration Rate, MDRD; Modification of Diet in Renal Disease, BIS-eGFR; Berlin Initiative Study-estimated Glomerular Filtration Rate, HDL-C; high-density lipoprotein cholesterol, LDL-C; low-density lipoprotein cholesterol.

Quantitative data are shown as median and quartiles and categorical variables are presented as counts and percentages.

### 3.1 Confounders of the association of kidney function with neurocognitive performance

#### 3.1.1 Cross-sectional analysis for global cognitive score at TILDA wave 1

The correlation matrix for the covariates included in the regression models can be found in Supplementary material online, *Figure S1*. *Table 2* shows cross-sectional estimates for kidney function markers (cystatin, CKD-EPI eGFRcys, and all eGFRcre formulas) at study Wave 1, for the entire study cohort and in separate analyses restricted to individuals without clinically significant CKD. The estimated VIFs suggested the absence of collinearity between variables in multivariable analyses. In univariable models, the estimate for the association of each kidney marker with lower cognitive performance was significant. In multivariable Model #1, these univariable associations were adjusted for age, and demographics (sex, education, and depression). Only cystatin C and CKD-EPI eGFRcys remained significant and were further included in the next multivariable models. In Model #2, adjusted for clinical vascular risk factors (together with age and demographics), the magnitude of coefficients for cystatin C and CKD-EPI eGFRcys was unaltered, while their effect was reduced after additional adjustment for inflammation and CVD biomarkers in Model #3. The pattern of the associations was similar in individuals with normal renal function compared to the whole study cohort and was not significant in the final Model #3.

**Table 2:** Results of linear regression analysis for the cross-sectional association of kidney function with global cognitive performance at wave 1

Regression Model	Univariable	Multivariable 1	Multivariable 2	Multivariable 3
		β-coefficient, SE (*P*-value)		
*Entire study population*				
Cystatin C	-0.36, 0.03 (*P* < 0.001)	-0.14, 0.03 (*P* < 0.001)	-0.14, 0.03 (*P* < 0.001)	-0.09, 0.03 (0.01)
Cystatin C-EPI-eGFR	0.38, 0.03 (*P* < 0.001)	0.13, 0.03 (*P* < 0.001)	0.12, 0.03 (*P* < 0.001)	0.08, 0.04 (0.03)
Creatinine-EPI-eGFR	0.25, 0.03 (*P* < 0.001)	0.04, 0.03 (*P* = 0.20)	n.a.	n.a.
Creatinine-BIS eGFR	0.32, 0.03 (*P* < 0.001)	0.00, 0.04 (*P* = 0.94)	n.a.	n.a.
MDRD	0.14, 0.03 (*P* < 0.001)	0.02, 0.03 (*P* = 0.51)	n.a.	n.a.
*eGFRcyscre > 60 mL/min*				
Cystatin C	-0.39, 0.05 (*P* < 0.001)	-0.15, 0.05 (*P* = 0.001)	-0.13, 0.05 (*P* = 0.007)	-0.08, 0.05 (0.08)
Cystatin C-EPI-eGFR	0.32, 0.04 (*P* < 0.001)	0.11, 0.04 (*P* = 0.004)	0.09, 0.04 (*P* = 0.01)	0.06, 0.04 (0.13)

**Abbreviations:** EPI-eGFR; Chronic Kidney Disease Epidemiology Collaboration-estimated Glomerular Filtration Rate, MDRD; Modification of Diet in Renal Disease, BIS-eGFR; Berlin Initiative Study-estimated Glomerular Filtration Rate, SE; standard error.

The standardized coefficient estimates were computed to compare the relative strength of the predictors within the regression models. N.a. indicates not assessed.

**Multivariable Model #1** was adjusted for age and demographics (i.e. sex, education, and depression).

**Multivariable Model #2** was adjusted for Multivariable Model 1, and clinical vascular risk factors (i.e. smoking, obesity, systolic blood pressure, diastolic blood pressure, number of CVD risk factors, diabetes mellitus, total cholesterol level, HDL-cholesterol level, C-reactive protein level).

**Multivariable Model #3** was adjusted for covariates from Multivariable Model 1, Model 2, and biomarkers (inflammation biomarker score, and cardiovascular biomarker score).

Pulse wave velocity was excluded from the analyses. Of note, the β-coefficient for cystatin C concentration (based on the analysis in the total study cohort) was -0.06 when PWV was forced among the covariates into the Multivariate Model #3. The Beta coefficients from the analysis restricted to the individuals without pulse wave velocity data were Beta = -0.26 (*P* < 0.001) in the univariable analysis, -0.10 (*P* = 0.006) in Multivariable Model #1, -0.08 (*P* = 0.03) for Multivariable Model #2 and -0.06 (*P* = 0.10) for Multivariable Model #3.

#### 3.1.2 Longitudinal analysis for cognitive decline

An analogous nested-adjusted scheme was used to model the risk of cognitive decline from Wave 1 to Wave 3, using a longitudinal linear mixed effect model. In univariable model baseline cystatin C, CKD-EPI eGFRcys, and all eGFRcre formulas were significantly associated with cognitive decline (*Table 3*). In comparison to the cross-sectional approach, the estimates were weaker in all models and were not significant in fully adjusted Model #3. In an additional analysis restricted to individuals without clinically significant CKD, the kidney markers lost significance after accounting for age and clinical covariates.

**Table 3:** Results of linear mixed effect model for the longitudinal association of kidney function with the decline in cognitive performance from TILDA wave 1 to wave 3

	Univariable	Multivariable 1	Multivariable 2	Multivariable 3
		β-coefficient, SE (*P*-value)		
*Entire study population*				
Cystatin C	-0.20, 0.01 (*P* < 0.001)	-0.07, 0.02 (*P* = 0.002)	-0.07, 0.02 (*P* = 0.003)	-0.01, 0.02 (*P* = 0.53)
Cystatin C-EPI-GFR	0.21, 0.01 (*P* < 0.001)	0.04, 0.02 (*P* = 0.08)	n.a.	n.a.
Creatinine-EPI-GFR	0.16, 0.02 (*P* < 0.001)	0.02, 0.02 (*P* = 0.43)	n.a.	n.a.
Creatinine-BIS GFR	0.20, 0.02 (*P* < 0.001)	-0.02, 0.02 (*P* = 0.25)	n.a.	n.a.
MDRD	0.08, 0.02 (*P* < 0.001)	0.00, 0.02 (*P* = 0.68)	n.a.	n.a.
*eGFRcyscre > 60 mL/min*				
Cystatin C	-0.13, 0.03, (*P* < 0.001)	-0.03, 0.03 (*P* = 0.38)	n.a.	n.a.
Cystatin C-EPI-GFR	0.13, 0.03 (*P* < 0.001)	0.03, 0.03(*P* = 0.30)	n.a.	n.a.

**Abbreviations:** EPI-eGFR; Chronic Kidney Disease Epidemiology Collaboration-estimated Glomerular Filtration Rate, MDRD; Modification of Diet in Renal Disease, BIS-eGFR; Berlin Initiative Study-estimated Glomerular Filtration Rate, n.a.; not assessed, SE; standard error.

The standardized coefficient estimates were computed to compare the relative strength of the predictors within the models.

**Multivariable Model #1** was adjusted for age and demographics (i.e. sex, education, and depression), and baseline cognitive score.

**Multivariable Model #2** was adjusted for Multivariable Model 1, and clinical vascular risk factors (i.e. smoking, obesity, systolic blood pressure, diastolic blood pressure, number of CVD risk factors, diabetes mellitus, total cholesterol level, HDL-cholesterol level, C-reactive protein level).

**Multivariable Model #3** was adjusted for Multivariable Model 1, Model 2, and biomarkers (inflammation biomarker score, and cardiovascular biomarker score).

Pulse wave velocity was excluded from the analyses. Of note, the β-coefficient for cystatin C concentration (based on the analysis in the total study cohort) was -0.00 when PWV was forced among the covariates into the Multivariate Model #3.

#### 3.1.3 Sensitivity analyses for cross-sectional and longitudinal associations with cognitive decline

As a sensitivity analysis, the same approaches were applied to participants without any CVD diseases and it showed a similar pattern and magnitude of associations between the same kidney markers in the regression Models #1, #2, and #3. The detailed results of sensitivity analyses for cognition level and cognitive decline are shown in Supplementary material online, *Table S2*.

### 3.2 Confounders of longitudinal association of kidney function with indices of cerebral vascular damage by brain imaging

**Figure 1:**
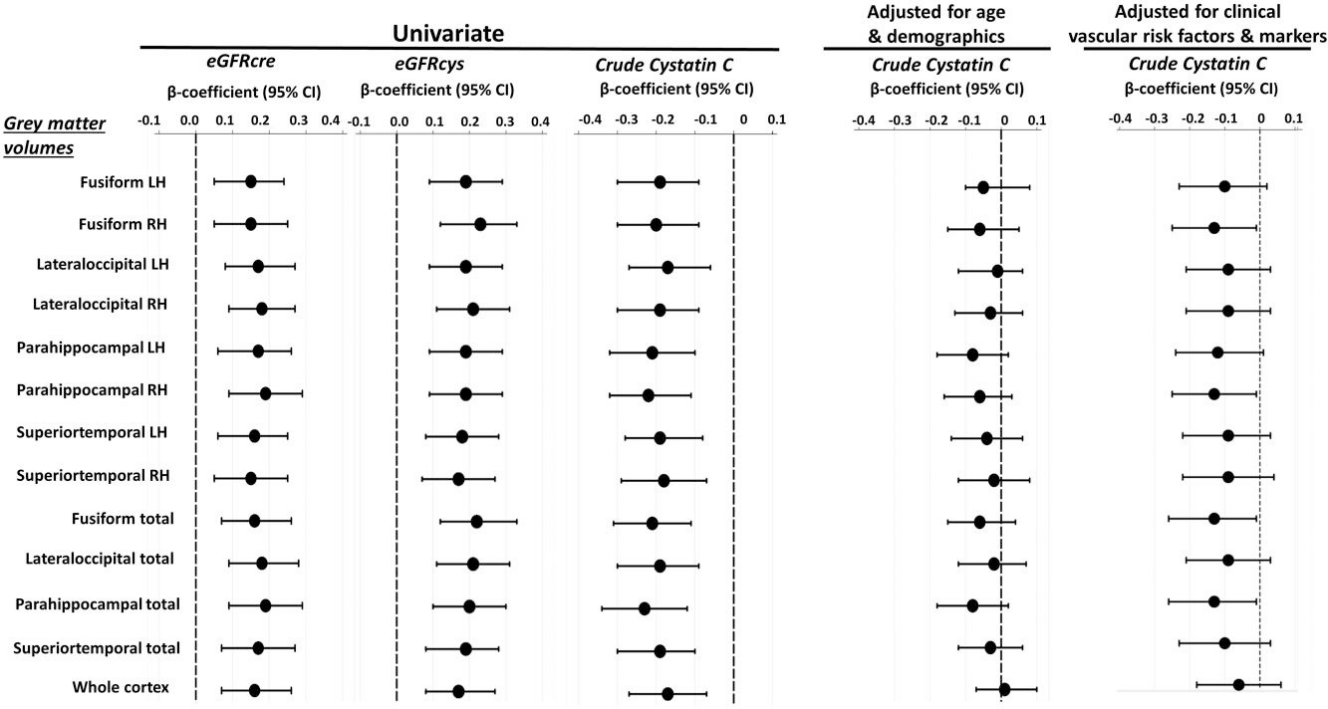
Results of linear regression analysis for grey matter volumes that were most significantly associated with kidney function markers. A total of 4 functional regions were significantly associated with kidney function in univariable analysis after accounting for multiple comparisons (see *Table 3*). Grey matter volumes were standardized for total intracranial volume. The grey matter volumes are presented for the left and right hemispheres and for both hemispheres combined. Standardized coefficients (βeta-estimates) and 95% confidence intervals (CIs) are presented per increase of *z*-score transformed volumes and each kidney marker, in univariable analysis and multivariable analysis adjusted for age and demographics (i.e. sex, education, depression), and in separate multivariable analysis adjusted for clinical vascular factors and biomarkers (i.e. smoking, obesity, systolic blood pressure, diastolic blood pressure, number of CVD risk factors, diabetes mellitus, total cholesterol level, HDL-C, CRP, pulse wave velocity, inflammation biomarker score, and cardiovascular biomarker score). Of note, based on the inspection of the changes in the cystatin-C/eGFRcys regression coefficients, age and cardiovascular BS were the most potent confounders of the association between kidney function and structural abnormalities in the respective regression models.

A total of 4 cortical regions were significantly associated with kidney function after accounting for multiple comparisons (see *Figure 1*). The β-coefficients are presented for grey matter volumes and each kidney marker (cystatin C, CKD-EPI eGFRcys, and CKD-EPI eGFRcre at Wave 1) in univariable models, and multivariable models adjusted for age and demographics, and in multivariable models adjusted for clinical vascular risk factors and biomarkers (see *Figure 1* and Supplementary material online, *Table S3*). In a multivariable model adjusted for age and demographics as well as in a separate multivariable model adjusted for clinical vascular risk factors and biomarkers, the Beta-estimates for all kidney markers became statistically not significant compared to the univariable approach (see Supplementary material online, *Table S3*). The graphical illustration of the comparison of the estimates from these models is shown in *Figure 1*.

In univariable regression analysis with scaled WMH as a dependant variable, cystatin C at Wave 1 (OR per 1 unit increase = 3.32 (95% CI, 1.11 to 9.98), and CKD-EPI eGFRcys at Wave 1 (OR per 1 unit increase = 0.98 (95% CI, 0.97 to 0.99) were significant predictors, whereas CKD-EPI eGFRcre at Wave 1 was borderline significant [OR per 1 unit increase = 0.99 (95% CI, 0.97 to 1.00)]. The associations were not present after adjusting for age and demographics or for clinical vascular risk factors and biomarkers. Similar results were obtained for kidney markers measured at Wave 3. The higher cystatin C quartile at Wave 1 displayed a markedly lower CBF_GM_ than the lower quartiles (Beta = -0.77 (95% CI, -0.05 to -1.49); however, this relationship flattened after adjustment in the multivariable models.

In additional analysis linking cystatin C with global function composite score; in participants included in the MRI dataset, the estimate was Beta = -0.55 (95% CI, -1.09 to 0.00) per 1 unit increase in the univariable model, and it was diminished after adjusting for total grey matter volume [Beta per 1 unit increase = -0.39 (95% CI, -0.96 to 0.15)].

### 3.3 Mediation analysis

*Table 4* Section A displays the results of our mediation assessing the relative contributions of each of the different covariates on the association between kidney function and global cognitive performance (at TILDA wave 1). We found that age was the predominant mediator of the association of cystatin C with cognitive dysfunction. However, beyond age, cardiovascular BS was another influential mediator of this association. Cardiovascular BS was a more powerful mediator of the association than inflammation BS. When assessing the association of cystatin C with the difference in cognitive scores over follow-up (between Wave 3 and Wave 1 TILDA) by mediation analysis (see *Table 4* Section B), the proportions of the mediated effect were small for the inflammation BS, and high for age and cardiovascular BS. Overall, this suggests that the effect of kidney function on the development of cognitive decline depends on arteriosclerosis markers on top of vascular ageing mechanisms.

**Table 4:** Results of mediation analysis for the association of cystatin C with A) global cognitive performance at Wave 1 and B) Change in the level of cognitive performance from Wave 1 to Wave 3: changes in the linear regression coefficient relating the association of cystatin C with global cognitive performance in multivariable regression models after separate entry of age, Cardiovascular BS and Inflammatory BS. Mediation analyses for cognitive domains are reported in Supplementary Material. Sensitivity analysis for participants without a history of CVD is in Supplementary Material

A. Mediation modelling for global cognition level	*Mediated effect per 1 unit increase in lncysC*	*P-value*	*% Coefficient reduction (CI)*	*P-value*
Univariable + Age	-0.89 (-1.06 - -1.73)	<0.001	46 (36–57)	<0.001
Univariable + Cardiovascular BS	-0.60 (-0.78 - -0.41)	<0.001	30 (20–40)	<0.001
Univariable + Inflammation BS	-0.05 (-0.10 - 0.00)	0.02	3 (0–4)	0.02
Multivariable#1* + Age	-0.63 (-0.77 - -0.49)	<0.001	47 (32–61)	<0.001
Multivariable#1 + Cardiovascular BS	-0.23 (-0.36 - -0.10)	<0.001	32 (9–54)	0.007
Multivariable#1 + Inflammation BS	-0.05 (-0.09 - -0.00)	0.02	7 (0–13)	0.04
Multivariable#2* + Age	-0.49 (-0.61 - -0.35)	<0.001	42 (27–58)	<0.001
Multivariable#2 + Cardiovascular BS	-0.23 (-0.36 - -0.09)	<0.001	33 (8–57)	0.009
Multivariable#2 + Inflammation BS	-0.04 (-0.08 - -0.01)	0.03	6 (-0–12)	0.06

B. Mediation modelling for cognitive decline^a^	*Mediated effect per 1 unit increase in lncysC*	*P-value*	*% Coefficient reduction (CI)*	*P-value*
Univariable + Age	-0.68 (-0.79 - -0.57)	<0.001	67 (50–85)	<0.001
Univariable + Cardiovascular BS	-0.58 (-0.77 - -0.54)	<0.001	57 (39–75)	<0.001
Univariable + Inflammation BS	-0.00 (-0.03 - 0.02)	0.69	1(-2–3)	0.69
Multivariable#1* + Age	-0.67 (-0.78 - -0.56)	<0.001	71 (52–91)	<0.001
Multivariable#1 + Cardiovascular BS	-0.29 (-0.39 - -0.18)	<0.001	99 (2–200)	0.04
Multivariable#1 + Inflammation BS	-0.01 (-0.03 - 0.01)	0.52	4 (-8–15)	0.50
Multivariable#2* + Age	-0.54(-0.65 - -0.34)	<0.001	38 (24–51)	<0.001
Multivariable#2 + Cardiovascular BS	-0.28 (-0.38 - -0.17)	<0.001	97 (5–180)	0.03
Multivariable#2 + Inflammation BS	-0.01 (-0.03 - 0.01)	0.51	3 (-6–12)	0.53

**Abbreviations:** BS; biomarker score, CI; 95% confidence interval, SD; standard deviation, ln; natural logarithm.

**Multivariable Model#1** was adjusted for age, and demographics (sex, education, depression)

**Multivariable Model#2** was adjusted for Multivariable Model#1 and clinical vascular risk factors (i.e. smoking, obesity, diabetes, number of CVD risk factors, systolic BP, diastolic BP, cholesterol, HDL cholesterol, CRP level)

Multivariable Model#1* was adjusted for demographics (sex, education, depression)

Multivariable Model#2* was adjusted for Multivariable Model#1* and clinical vascular risk factors (smoking, obesity, diabetes, number of CVD risk factors, systolic BP, diastolic BP, cholesterol, HDL cholesterol, CRP level)

Longitudinal models for cognitive decline were additionally adjusted for baseline cognitive score.

Systolic blood pressure, diastolic blood pressure, C-reactive protein were not significant mediators, and as such the mediation results are not shown.

**Figure 2:**
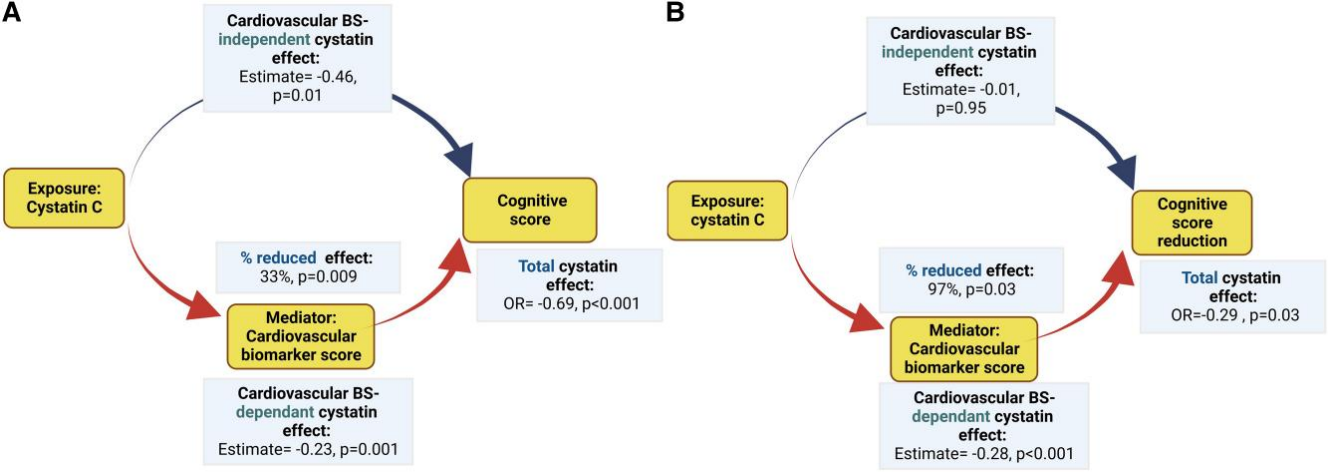
Purpose of mediation used in the study. Directed acyclic graph theorizing possible etiological relationships. The graph is not exhaustive. The effect of cystatin C on neurocognitive abnormalities (global cognition at TILDA Wave 1, cognitive score reduction between Wave 1 and Wave 3) in the study cohort is exercised as an example. The estimates represent the total and decomposed effects of kidney function in a one mg/mL unit increase of plasma cystatin C transformed to its base natural logarithms (ln-cystatin C). A cardiovascular biomarker score (Cardiovascular BS) is tested as a mediator. The analyses were based on a multivariable linear regression model adjusted for age, demographics (sex, education, depression), and clinical vascular risk factors (i.e. smoking, obesity, systolic blood pressure, diastolic blood pressure, number of CVD risk factors, diabetes mellitus, total cholesterol level, HDL-cholesterol level, C-reactive protein level). Mediation results for vascular markers are described in *Table 4* (Panels A and B).

*Figure 2* outlines the conceptual framework of mediation for the cardiovascular BS, based on linear regression model adjusted for relevant covariates. Panel A illustrates mediation results for global cognitive function at TILDA Wave 1. The effect of cystatin on global cognitive function (total effect) is split into a natural indirect effect, which exerts its role on the cognitive outcomes through modulation of cystatin C by cardiovascular biomarkers, and a direct effect, which acts on the outcome independently from the mediator. The total effect of cystatin on global cognitive score was -0.69 (95% CI, -1.02 to -0.37). Cystatin C had significant cardiovascular BS-dependant (-0.23 per 1 lncysC increase, *P* < 0.001) and direct (-0.46 per 1 lncysC increase, *P* = 0.01) effects. The proportion of mediated effect was 33% (*P* = 0.009). Similar analysis was performed to examine the association of global cognitive function loss (between Wave 1 and Wave 3) with cystatin C, using mediation analysis (panel B). Consequently, the total cystatin C effect on global cognitive decline was -0.29 per 1 lncysC increase, the cardiovascular BS-dependant cystatin C effect was -0.28 per 1 lncysC increase, and the proportion of the mediated cystatin C effect was 97% (*P* = 0.03). Each of these effects was statistically significant. The direct cystatin C effect was not statistically significant (panel B).

The results of sensitivity analyses for the same mediators and global cognitive function and decline can be found in Supplementary material online, *Table S4*. The mediated effects decreased only moderately when participants without CVD conditions were considered.

### 3.4 Secondary analyses of cognitive domains

**Figure 3:**
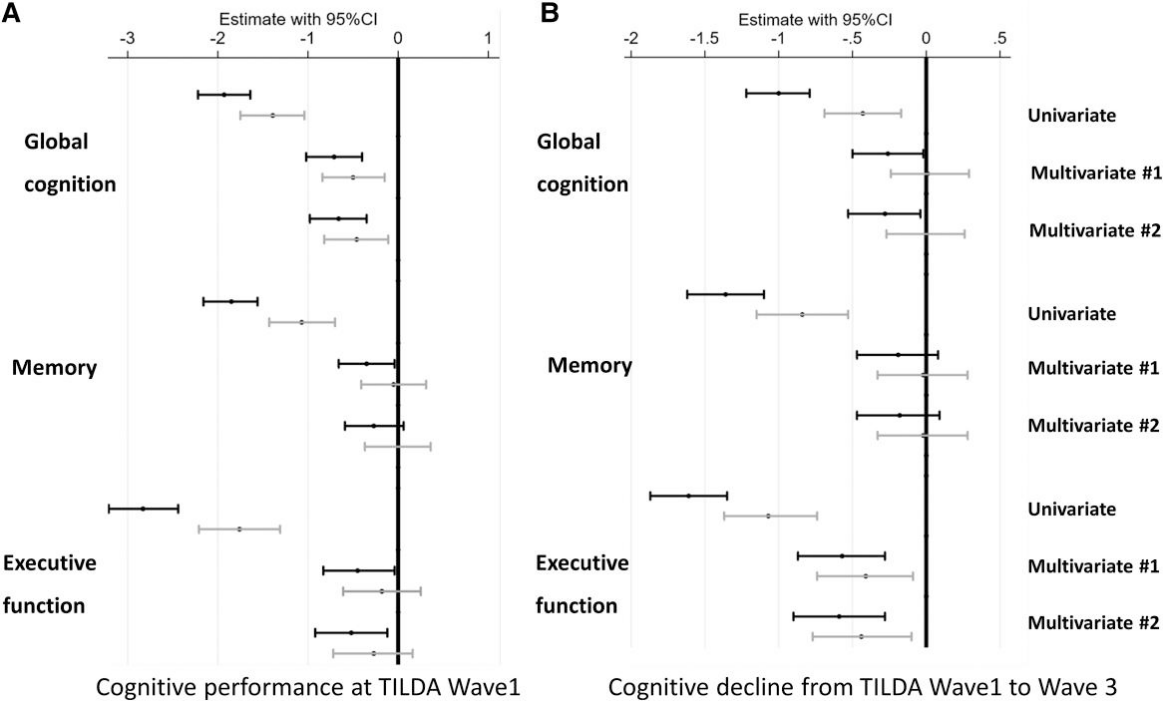
Vascular score-independent cystatin c-effects (black confidence interval) and vascular biomarker score-adjusted (grey confidence interval) cystatin c-effects on global cognition, memory, and executive function/attention performance and their decline in the univariable and multivariable regression models. Multivariable Model #1 was adjusted for age and demographics (sex, education, and depression). Multivariable Model #2 was adjusted for Multivariable Model 1, and clinical vascular risk factors (i.e. smoking, obesity, systolic blood pressure, diastolic blood pressure, number of CVD risk factors, diabetes mellitus, total cholesterol level, HDL-cholesterol level, C-reactive protein level). Longitudinal models were additionally adjusted for baseline cognitive score.

The results were consistent when other cognitive domains (memory, and executive function) were considered as an outcome. A plot of total and adjusted cystatin C effects for each cognitive domain (baseline or the difference between study waves) is shown in *Figure 3* and the results of mediation analyses for age, inflammation BS, and cardiovascular BS are displayed in Supplementary material online, *Tables S5–S8*.

## 4. Discussion

In this study, we demonstrate, as first, the significance of potential mediating mechanisms, such as age and cardiovascular risk biomarkers, in characterizing the association of normal to mildly decreased eGFR, cognitive performance level, and the subsequent risk of cognitive decline. Additionally, in our MRI sub-study, the associations between kidney function and indices of structural brain damage, such as CBF_GM_, global WMH, and grey matter volume, were strongly significant in univariable models and lost significance after adjusting for confounding factors.

Although the association between kidney function and neuro-cognitive function likely represents overlapping complex processes, given their contribution in our analysis suggests that age and CVD biomarkers are the strong mediators of this association. An alternative potential mechanism linking kidney dysfunction with the risk of cognitive impairment posits that the alterations in concentrations of potentially toxic compounds in response to decline in kidney function may directly contribute to neuron injury and accelerated cognitive loss.^3^ It was documented previously that the clearance of uremic solutes predicts cognitive decline in mild-to-moderate CKD.^31^ In addition, implemented Mendelian randomization approaches suggested that genetically predicted kidney glomerular damage might have casually influenced cortical thickness.^32^ From our findings, we can consider progression to dementia and cognitive loss related to reduced eGFR, primarily due to aging and arteriosclerosis processes.

We identified two sets of mediators representing vascular aging and arteriosclerosis, and they overlapped only partially. Regarding the first mechanism, we were able to retrieve one, recent observation, highlighting age as a potential confounder of a relationship between eGFRcre and brain atrophy.^33^ On the other hand, cardiovascular BS, constructed from the combined levels of GDF15 and NT-proBNP, was another significant mediator identified by us, and there is increasing literature regarding the involvement of these proteins in arteriosclerotic processes. GDF15 is a member of TGF-βeta superfamily, which is important in the early and acute inflammatory stages leading to vulnerable plaque. GDF15 was detected in the prostate, placenta, heart, intestine, liver, kidney, pancreas, lung, and brain and it is likely to play a role in a wide range of physiological processes. While its expression is wildly distributed across different tissues, GDF15 is highly expressed in vascular endothelial cells and is induced during ischemia to promote angiogenesis.^34^ Both GDF15 and NT-proBNP were similarly upregulated by stress in isolated cardiomyocytes and in mice hearts.^34^ Multiple studies showed that elevated circulating levels of these markers are independently associated with an elevated risk of Coronary Artery Disease.^34^ A combination of GDF15 and NT-proBNP has recently been reported to predict incident cognitive dysfunction in the Framingham cohort.^35^ Our study takes these associations further to explore the possibility that GDF15 may be a useful candidate biomarker of cognitive dysfunction as it relates to eGFR in community-dwelling individuals, perhaps mediated through cerebral atherosclerosis, and as a functional marker of CVD risk since GDF15 is known to be associated with subclinical atherosclerosis.^34^ While it is tempting to assume causal subclinical CVD pathways aligning GDF15 and NT-proBNP levels with simultaneous reductions in kidney function and cerebral function we must temper such assumptions by acknowledging the possibility of residual confounding by other comorbidities or factors associated with these biomarkers. In addition, given that our findings were gleaned from a population-representative sample, while it is possible that our findings may have clinical significance, we are unable to discern relevant contributions to clinical significance rather than mere statistical significance. Further study is required to investigate the potential of biomarkers such as GDF15 and NT-proBNP for discriminating risk of cognitive impairment in association with CKD.

Two other results from our study require mentioning. Firstly, consistent with previous findings we observed that executive function could be more affected by eGFR, at early CKD stage, than other domains, and we further complement this result with evidence of mediation by vascular processes. Secondly, of multiple grey matter areas examined by us in univariable analyses, the hippocampal and temporal areas were strongly associated with kidney function. These are highly metabolically demanding regions that rely on oxygen supply, as reported by previous studies on vascular stiffness in cognition.^36^ The characteristics and congruence of the determinants of eGFR are further evidence for our hypothesis that vascular dysfunction is the primary process underlying kidney disease and the development of cognitive decline, in people with relatively preserved kidney function.

Our study adds to previously published research concerning cognition and kidney dysfunction.^6–8,37–40^ These studies often focused on very elderly populations with moderate or severe CKD, they utilized a single eGFR equation, and most importantly none of them explored the specific factors contributing to the association of kidney dysfunction with neuropsychological degeneration. While our study has multiple strengths (including large, population-based, prospective study design, multiple eGFR formulas, comprehensive adjusting in multivariable models, and consistency of analysis across a spectrum of outcomes) we also need to acknowledge its limitations. This study is observational, and causality needs to be established through animal studies and clinical trials; however, mediation analysis supports our hypothesis. Another limitation is a shorter follow-up time than needed to develop clinically significant dementia. Since TILDA was not specifically designed to evaluate the effect of kidney dysfunction on cognition, other indices of kidney impairment, such as albuminuria, were not collected. Further, while GDF15 may be a marker of subclinical atherosclerosis or endothelial damage, this study was limited in the assessment of endothelial damage due to the lack of availability of von Willebrand factor levels.^41,42^ Another limitation of CVD biomarker assessments in biological samples lies in the considerable diurnal variation in their concentrations, as reported by previous studies.^43^ As stated previously, ours was a single-study, with an observational sample design, and thus our results need to be considered hypothesis-generating.

In community-dwelling individuals, the association between kidney function, cognitive function, and cognitive function loss appears to be predominantly mediated by age and cardiovascular risk biomarkers. The detailed mechanistic effects of those biomarkers in GFR related cognitive decline needs to be established through animal studies and clinical trials. The TILDA MRI brain structural data reveal that whereas kidney function markers were associated with white matter disease, and with decreased grey matter volumes within the areas susceptible to hypoxia, these associations were diminished by age or by the combination of clinical vascular factors and biomarkers. The observation that cardiovascular marker clinical use could aid cognitive risk assessment is encouraging, but more research is needed to establish whether the exemplars of GDF15 or combinations of biomarkers could potentially aid in identifying individuals at particular risk for cognitive decline in whom drug treatments, intensive cardiovascular risk factor modification, and early cognitive interventions may be helpful. Together, our findings lend credence to the concept that the concomitant decline in cognitive function and kidney function with ageing in the general population may be predominately mediated by subclinical or overt vascular disease processes manifesting in two separate organs which are highly reliant on blood vessel health.

## Supplementary material

Supplementary material is available at *Cardiovascular Research* online.

## Authors’ contributions

N.N. (Ph.D.), C.D.L. (Ph.D.), A.O.H. (Ph.D.), R.A.K. (MD Ph.D.), and D.S. (MD Ph.D.)

## Supplementary Material

cvad060_Supplementary_Data

## Data Availability

Data from the TILDA study are publicly available at the Irish Social Science Data Archive based in University College Dublin, and the Interuniversity Consortium for Political and Social Research (ICPSR) based in the University of Michigan. The privileged TILDA datasets are available only on justified request.

## Notes

Translational perspective In our prospective cohort study, age, and concentrations of biomarkers previously related to CVD were found to be mediators of the association between mild to moderately decreased kidney function and neurocognitive abnormalities. In particular, a score composed of combined levels of GDF15 and NT-proBNP was associated with cystatin C; and was mediating the adjusted association between kidney function and cognitive decline. Inflammation biomarker score showed borderline mediating effect. These results suggest potential causal vascular pathways that underline both development of early kidney function decline and cognitive decline. Additionally, CVD biomarkers could be evaluated in discriminating individuals at particular risk of dementia in the general population, who may benefit from intensive CVD risk factor modification.
