# Supplementary material for: The association between kidney function, cognitive function, and structural brain abnormalities in community-dwelling individuals aged 50+ is mediated by age and biomarkers of cardiovascular disease
Source: Cardiovasc Res. 2023 Apr 13;119(11):2106–16. doi: 10.1093/cvr/cvad060 (PMC10683948; doi:10.1093/cvr/cvad060)
Supplement: cvad060_Supplementary_Data [file cvad060_supplementary_data.zip › Cardiovascular Research_Supplementary Material.270123.docx]

**27/01/2023**

**Supplementary material**

**Title: The association between kidney function, cognitive function, and structural brain abnormalities in community-dwelling individuals aged 50+ is mediated by age and biomarkers of cardiovascular disease**

**Running Title:** Vascular factors attenuate GFR-related brain abnormalities

Natalia Nowak PhD^1,2^, Celine De Looze PhD^1,2^, Aisling O'Halloran PhD^1,2^, Rose Anne Kenny MD, PhD^1,2^, Donal J Sexton MD, PhD^1,2,3^

^1^ The Irish Longitudinal Study on Ageing, Trinity College Dublin, Ireland.

^2^ School of Medicine, Trinity College Dublin, Ireland

^3^ Division of Nephrology, Saint James Hospital, Dublin, Ireland

**Address for correspondence:**

Natalia Nowak, PhD

School of Medicine,

Trinity College Dublin

St James's Hospital

James Street Dublin 8

Ireland D08 NHY1

e-mail: nowakn@tcd.ie

nzuznowak@gmail.com

**Supplementary Tables and Figures**

**Table of Contents:**

**Supplementary Methods**

**Supplementary Table S1.** Cognitive performance by quartiles of cystatin C distribution at TILDA Wave 1 and Wave 3

**Supplementary Figure S1.** Spearman rank correlation analysis for cystatin C and the covariates included in the multivariable analyses for cognitive performance

**Supplementary Table S2.** Sensitivity analyses for the association of kidney function with global cognitive performance and cognitive decline: analyses restricted to participants without a history of any cardiovascular conditions (angina, stroke, TIA, heart failure, or heart attack (including myocardial infarction or coronary thrombosis)

**Supplementary Table S3.**  Unadjusted and adjusted association between kidney function and brain volumes

**Supplementary Table S4.** Sensitivity analysis: mediation analyses restricted to participants without history of any cardiovascular conditions

**Supplementary Table S5.** Mediation analysis for the association of cystatin C with memory

**Supplementary Table S6.** Mediation analysis for the association of cystatin C with executive function

**Supplementary Table S7.** Mediation analysis for the association of cystatin C with the difference in memory between Wave 1 and Wave 3

**Supplementary Table S8.** Mediation analysis for the association of cystatin C with the difference in executive function between Wave 1 and Wave 3

**Supplementary Methods**

The assays used in TILDA study have the validation and Q&C parameters determined by the manufacturer. Inter-assay variability reflected as coefficients of variation (CV) was based on the measurement of our technical replicates (two samples were repeated on each plate across 62 plates for the TILDA W1 plasma samples (248 replicates or 124 replicate pairs)).

| **Biomarker** | **CV (%)** |
| --- | --- |
| N-terminal pro-B-type natriuretic peptide (NT-proBNP) | 6.80 |
| Growth Differentiation Factor 15 (GDF15) | 9.24 |
| Interleukin-1 Receptor Antagonist (L1RA) | 9.54 |
| Interleukin-6 (IL-6) | 8.06 |
| Interleukin-8 (IL-8) | 4.29 |
| Tumor Necrosis Factor-alpha (TNFα) | 6.10 |

**Supplementary Table S1.** Cognitive performance by quartiles of cystatin C distribution at Wave 1 and Wave 3

| ***Wave 1 Cystatin C*** | **1st quartile** | **2nd quartile** | **3^rd^ quartile** | **4^th^ quartile** | **p-value** |
| --- | --- | --- | --- | --- | --- |
| ***Global cognition*** |  |  |  |  |  |
| MOCA | 26.0 (2.8) | 25.6. (3.1) | 25.3 (3.1) | 24.3 (3.5) | <0.001 |
| MMSE | 29.0 (1.6) | 28.7 (1.8) | 28.5 (1.6) | 28.3 (2.0) | <0.001 |
| ***Memory*** |  |  |  |  |  |
| Immediate recall | 14.4 (2.8) | 14.1 (2.8) | 13.6 (3.0) | 13.0 (3.1) | <0.001 |
| Delayed recall | 6.8 (2.1) | 6.5 (2.2) | 6.2 (2.2) | 5.8 (2.2) | <0.001 |
| ***Executive function/attention*** |  |  |  |  |  |
| Colour trails time 1 | 49.4 (19.6) | 52.4 (23.9) | 53.7 (21.6) | 62.7 (28.6) | <0.001 |
| Colour trails time 2 | 99.2 (33.1) | 103.4 (34.8) | 109 (37.6) | 120.5 (44.3) | <0.001 |
| Mean SART time | 376.7 (93.0) | 379.5 (94.5) | 385.2 (94.2) | 399.3 (96.9) | <0.001 |
| ***Wave 3 Cystatin***  ***Global cognition*** | **1st quartile** | **2nd quartile** | **3rd quartile** | **4th quartile** | **p-value** |
| MOCA | 26.3 (3.0) | 26.0 (3.1) | 25.6 (3.3) | 24.3 (4.2) | <0.001 |
| MMSE | 29.1 (1.4) | 28.9 (1.5) | 28.8 (1.5) | 28.2 (2.0) | <0.001 |
| ***Memory*** |  |  |  |  |  |
| Immediate recall | 14.6 (2.7) | 14.2 (2.9) | 14.0 (2.9) | 12.8 (3.1) | <0.001 |
| Delayed recall | 6.7 (2.2) | 6.4 (2.4) | 6.2 (2.3) | 5.4 (2.5) | <0.001 |
| ***Executive function/attention*** |  |  |  |  |  |
| Colour trails time 1 | 49.4 (21.3) | 52.4 (25.2) | 55.7 (24.3) | 67.9 (37.0) | <0.001 |
| Colour trails time 2 | 99.6 (33.7) | 103.5 (38.7) | 111.9 (41.0) | 128.6 (52.3) | <0.001 |
| Mean SART time | 340.4 (81.0) | 346.2 (89.5) | 350.2 (80.8) | 383.7 (96.5) | <0.001 |

Abbreviations: MOCA; Montreal Cognitive Assessment, MMSE; Mini-Mental State Exam (MMSE), SART; Sustained Attention to Response Task Test.

The data are shown as mean and standard deviation. P-values are from the Jonckheere-Terpstra Trend Test.

**Supplementary Figure S1.** Spearman rank correlation analysis for cystatin C and the covariates included in the multivariable analyses for cognitive performance

|  | **CYS** | **AGE** | **CVD-BS** | **PWV** | **INFLAM**  **-BS** | **CRP** | **BMI** | **CHOL** | **HDL** | **SYS**  **BP** | **DIA**  **BP** |
| --- | --- | --- | --- | --- | --- | --- | --- | --- | --- | --- | --- |
| CYS C | 1 | 0.43 | 0.49 | 0.29 | 0.27 | 0.27 | 0.22 | -0.16 | -0.2 | 0.14 | 0.01 |
| AGE |  | 1 | 0.53 | 0.51 | 0.14 | 0.09 | 0.02 | -0.19 | -0.05 | 0.27 | -0.05 |
| CVD-BS |  |  | 1 | 0.32 | 0.29 | 0.19 | 0.09 | -0.12 | -0.1 | 0.17 | -0.04 |
| PWV |  |  |  | 1 | 0.16 | 0.12 | 0.14 | -0.12 | -0.16 | 0.42 | 0.20 |
| Inflamm-BS |  |  |  |  | 1 | 0.25 | 0.21 | -0.05 | -0.2 | 0.14 | 0.10 |
| CRP |  |  |  |  |  | 1 | 0.34 | 0.02 | -0.14 | 0.08 | 0.08 |
| BMI |  |  |  |  |  |  | 1 | -0.12 | -0.34 | 0.15 | 0.18 |
| CHOL |  |  |  |  |  |  |  | 1 | 0.41 | -0.01 | 0.12 |
| HDL |  |  |  |  |  |  |  |  | 1 | -0.07 | -0.02 |
| SysBP |  |  |  |  |  |  |  |  |  | 1 | 0.74 |
| DiaBP |  |  |  |  |  |  |  |  |  |  | 1 |

**Abbreviations:** BS; biomarker score, CRP; C-reactive protein, CVD; cardiovascular, Chol; Total cholesterol, DIA BP; diastolic blood pressure, HDL; high-density lipoprotein cholesterol, SYS BP; systolic blood pressure, PWV; pulse wave velocity.

**Supplementary Table S2.** Sensitivity analyses for the association of kidney function with global cognitive performance and cognitive decline. Analyses were restricted to participants without history of any cardiovascular conditions (angina, stroke, TIA, heart failure, or heart attack (including myocardial infarction or coronary thrombosis)

**Regression Model Univariable Multivariable 1 Multivariable 2 Multivariable 3**

**β-coefficient (95% CI)**

***Global cognition at Wave 1***

**Cystatin C** -0.38 (-0.44, -0.14) -0.15 (-0.21, -0.09) -0.14 (-0.20, -0.07) -0.09 (-0.16, -0.02)

**Cystatin C-EPI-eGFR** 0.38 (0.32, 0.43) 0.14 (0.07, 0.20) 0.12 (0.06, 0.19) 0.08 (0.00, 0.15)

**Creatinine-EPI-eGFR** 0.22 (0.16, 0.28) 0.01 (-0.04, 0.07) n.a. n.a.

**Creatinine-BIS eGFR** 0.29 (0.23, 0.35) -0.01 (-0.08, 0.06) n.a. n.a.

**MDRD**  0.11 (0.05, 0.17) 0.00 (-0.06, 0.05) n.a. n.a.

***Cognitive decline from W1 to W3****

**Cystatin C** -0.18 (-0.21, -0.14) -0.05 (-0.09, -0.01) -0.06 (-0.09, -0.02) -0.03 (-0.02, 0.09)

**Cystatin C-EPI-GFR** 0.18 (0.13, 0.22) 0.02 (-0.03, 0.07 ) n.a. n.a.

**Creatinine-EPI-GFR** 0.16 (0.12, 0.20) 0.02 (-0.03, 0.06) n.a. n.a.

**Creatinine-BIS GFR** 0.20 (0.16, 0.24) -0.02 (-0.08, 0.02) n.a. n.a.

**MDRD**  0.08 (0.04, 0.12) 0.00 (-0.05, 0.03) n.a. n.a.

**Abbreviations:** EPI-eGFR; Chronic Kidney Disease Epidemiology Collaboration- estimated Glomerular Filtration Rate, MDRD; Modification of Diet in Renal Disease, BIS-eGFR; Berlin Initiative Study-estimated Glomerular Filtration Rate, CI; confidence interval.

The standardized coefficient estimates were computed to compare the relative strength of the predictors within the regression models. N.a. indicates not assessed.

**Multivariable Model #1** was adjusted for age and demographics (i.e., sex, education, and depression).

**Multivariable Model #2** was adjusted for Multivariate Model 1, and clinical vascular risk factors (i.e., smoking, obesity, systolic blood pressure, diastolic blood pressure, number of CVD risk factors, diabetes mellitus, total cholesterol level, HDL-cholesterol level, C-reactive protein level).

**Multivariable Model #3** was adjusted for covariates from Multivariate Model 1, Model 2, and biomarkers (inflammation biomarker score, and cardiovascular biomarker score).

*All longitudinal models were additionally adjusted for baseline cognitive score.

**Supplementary Table S3.** Unadjusted and adjusted association between kidney function and total regional brain volumes, and regional brain volumes within hemispheres (n=422)

|  | **Cystatin C**  *Beta (95%CI), p-value* | **CKD-EPI eGFRcys**  *Beta (95%CI), p-value* | **CKD-EPI eGFRcre**  *Beta (95% CI), p-value* |
| --- | --- | --- | --- |
| ***Unadjusted model*** | | | |
| **R Fusiform** | -0.20 (-0.30, -0.09), <0.001 | 0.23 (0.12, 0.33), <0.001 | 0.15 (0.05, 0.25), 0.002 |
| **L Fusiform** | -0.19 (-0.30, -0.09), <0.001 | 0.19 (0.09, 0.29), <0.001 | 0.15 (0.05, 0.25), 0.002 |
| **R Lateraloccipital** | -0.19 (-0.30, -0.09), <0.001 | 0.21 (0.11, 0.31), <0.001 | 0.18 (0.08, 0.27), <0.001 |
| **L Lateraloccipital** | -0.17 (-0.27, -0.06), 0.001 | 0.19 (0.09, 0.29), <0.001 | 0.17 (0.07, 0.27), <0.001 |
| **R Parahippocampal** | -0.22 (-0.32, -0.11), <0.001 | 0.19 (0.09, 0.29), <0.001 | 0.19 (0.09, 0.29), <0.001 |
| **L Parahippocampal** | -0.21 (-0.32, -0.10), <0.001 | 0.19 (0.09, 0.29), <0.001 | 0.17 (0.07, 0.27), <0.001 |
| **L Superiortemporal** | -0.19 (-0.28, -0.08), <0.001 | 0.18 (0.08, 0.28), <0.001 | 0.16 (0.06, 0.25), 0.001 |
| **R Superiortemporal** | -0.18 (-0.29, -0.07), <0.001 | 0.17 (0.07, 0.27), 0.002 | 0.15 (0.05, 0.25), 0.003 |
| **Fusiform** | -0.21 (-0.31, -0.11), <0.001 | 0.22 (0.12, 0.33), <0.001 | 0.16 (0.07, 0.26), <0.001 |
| **Lateraloccipital** | -0.19 (-0.30, -0.09), <0.001 | 0.21 (0.11, 0.31), <0.001 | 0.18 (0.09, 0.28), <0.001 |
| **Parahippocampal** | -0.23 (-0.34, -0.12), <0.001 | 0.20 (0.10, 0.30), <0.001 | 0.19 (0.09, 0.29), <0.001 |
| **Superiortemporal** | -0.19 (-0.30, -0.10), <0.001 | 0.19 (0.08, 0.28), <0.001 | 0.17 (0.07, 0.27), <0.001 |
| **Whole cortex** | -0.17 (-0.27, -0.07), 0.001 | 0.17 (0.07, 0.27), <0.001 | 0.16 (0.07, 0.26), 0.001 |
| ***Multivariable model adjusted for age and demographics*** | | | |
| **R Fusiform** | -0.06 (-0.15, 0.05), 0.28 | 0.04 (-0.06, 0.14), 0.43 | -0.01 (-0.10, 0.08), 0.76 |
| **L Fusiform** | -0.05 (-0.10, 0.08), 0.37 | 0.02 (-0.09, 0.12), 0.76 | -0.00 (-0.10, 0.08), 0.95 |
| **R Lateraloccipital** | -0.03 (-0.13; 0.06), 0.48 | 0.02 (-0.08, 0.11), 0.72 | 0.01 (-0.08, 0.08), 0.73 |
| **L Lateraloccipital** | -0.01 (-0.12, 0.06), 0.86 | 0.00 (-0.10, 0.09), 0.92 | 0.00 (-0.09, 0.09), 0.94 |
| **R Parahippocampal** | -0.06 (-0.16, 0.04), 0.24 | 0.06 (-0.04, 0.16), 0.23 | 0.05 (-0.02, 0.16), 0.14 |
| **L Parahippocampal** | -0.08 (-0.18, 0.02), 0.12 | 0.04 (-0.06, 0.14), 0.43 | 0.04 (-0.05, 0.14), 0.36 |
| **L Superiortemporal** | -0.04 (-0.14, 0.06), 0.44 | 0.01 (-0.08, 0.11), 0.77 | 0.02 (-0.06, 0.11), 0.56 |
| **R Superiortemporal** | -0.02 (-0.12, 0.08), 0.69 | 0.00 (-0.10, 0.09), 0.85 | 0.01 (-0.08, 0.10), 0.91 |
| **Fusiform** | -0.06 (-0.15, 0.04), 0.28 | 0.03 (-0.06, 0.13), 0.56 | -0.01 (-0.10, 0.08), 0.85 |
| **Lateraloccipital** | -0.02 (-0.12, 0.07), 0.62 | 0.01 (-0.08, 0.10), 0.87 | 0.00 (-0.08, 0.09), 0.82 |
| **Parahippocampal** | -0.08 (-0.18, 0.02), 0.14 | 0.06 (-0.04, 0.16), 0.28 | 0.06 (-0.03, 0.13), 0.20 |
| **Superiortemporal** | -0.03 (-0.12, 0.06), 0.51 | 0.01 (-0.09, 0.10), 0.94 | 0.02 (-0.06, 0.11), 0.71 |
| **Whole cortex** | 0.01 (-0.07, 0.10), 0.76 | -0.03 (-0.13, 0.05), 0.44 | -0.01 (-0.10, 0.06), 0.84 |
| ***Multivariable model adjusted for clinical vascular risk factors and biomarkers*** | | | |
| **R Fusiform** | -0.13 (-0.26, -0.01), 0.03 | 0.15 (0.05; 0.27), 0.006 | 0.08 (-0.01, 0.19), 0.10 |
| **L Fusiform** | -0.11 (-0.24, 0.02), 0.10 | 0.13 (0.02; 0.24), 0.03 | 0.09 (-0.02, 0.20), 0.10 |
| **R Lateraloccipital** | -0.09 (-0.21, 0.03), 0.17 | 0.10 (-0.00, 0.21), 0.07 | 0.10 (-0.01, 0.20), 0.06 |
| **L Lateraloccipital** | -0.08 (-0.21, 0.03), 0.15 | 0.10 (-0.00, 0.21), 0.07 | 0.10 (-0.01, 0.20), 0.06 |
| **R Parahippocampal** | -0.13 (-0.27, -0.01), 0.03 | 0.10 (-0.01, 0.21), 0.08 | 0.14 (0.04, 0.25), 0.01 |
| **L Parahippocampal** | -0.12 (-0.25, 0.01), 0.06 | 0.09 (-0.02, 0.20), 0.13 | 0.12 (0.01, 0.22), 0.02 |
| **L Superiortemporal** | -0.09 (-0.22, 0.03), 0.12 | 0.08 (-0.03, 0.20), 0.14 | 0.11 (0.01, 0.22), 0.04 |
| **R Superiortemporal** | -0.09 (-0.22, 0.04), 0.16 | 0.07 (-0.04, 0.19), 0.22 | 0.12 (0.02, 0.22), 0.04 |
| **Fusiform** | -0.13 (-0.26, -0.01), 0.04 | 0.15 (0.04, 0.27), 0.01 | 0.09 (-0.01, 0.20), 0.09 |
| **Lateraloccipital** | -0.09 (-0.21, 0.03), 0.13 | 0.11 (0.00, 0.21), 0.05 | 0.10 (0.01, 0.20), 0.05 |
| **Parahippocampal** | -0.13 (-0.26, -0.01), 0.03 | 0.10 (-0.01, 0.22), 0.07 | 0.15 (0.04, 0.24), 0.01 |
| **Superiortemporal** | -0.10 (-0.23, 0.03), 0.10 | 0.08 (-0.03, 0.20), 0.15 | 0.12 (0.01, 0.23), 0.03 |
| **Whole cortex** | -0.06 (-0.18, 0.06), 0.30 | 0.06 (-0.05, 0.17), 0.29 | 0.10 (0.00, 0.20), 0.04 |

**Abbreviations:** cys; cystatin C, cre; creatinine; EPI-eGFR; Chronic Kidney Disease Epidemiology Collaboration- estimated Glomerular Filtration Rate; CI; confidence interval. R and L indicate the right and left hemispheres.

**Supplementary Table S4.** Sensitivity analysis restricted to participants without a history of any cardiovascular conditions. Results of mediation analysis for the association of cystatin C with (A) global cognitive performance at TILDA Wave 1, and (B) Difference in cognitive performance from TILDA Wave 1 to Wave 3. Changes in the linear regression coefficient relating the association of cystatin C with cognitive outcomes in multivariable regression models after separate entries of each vascular marker

| 1. **Mediation modeling for global cognition level** | ***Mediated effect***  ***per 1unit increase in lncys*** | ***p-value*** | ***% Coefficient reduction (CI)*** | ***p-value*** |
| --- | --- | --- | --- | --- |
| Univariable + Age | -0.87 (-1.03- -0.71) | <0.001 | 45 (34 – 55) | <0.001 |
| Univariable+ Cardiovascular BS | -0.64 (-0.82 - -0.45) | <0.001 | 32 (21 – 42) | <0.001 |
| Univariable + Inflammation BS | -0.06 (-0.10 –0.01) | 0.02 | 3 (1 – 5) | 0.02 |
|  |  |  |  |  |
| Multivariable#1*+ Age | -0.62 (-0.76 - -0.48) | <0.001 | 45 (31 – 65) | <0.001 |
| Multivariable#1+ Cardiovascular BS | -0.25 (-0.38- -0.12) | <0.001 | 33 (10 – 55) | 0.004 |
| Multivariable#1+ Inflammation BS | -0.05 (-0.09- -0.01) | 0.02 | 7 (1 – 14) | 0.03 |
|  |  |  |  |  |
| Multivariable#2*+ Age | -0.47 (-0.61 - -0.34) | <0.001 | 41 (25 – 57) | <0.001 |
| Multivariable#2+Cardiovascular BS | -0.24 (-0.36- -0.12) | <0.001 | 33 (8 – 58) | 0.009 |
| Multivariable#2+ Inflammation BS | -0.04 (-0.09 - -0.01) | 0.04 | 6 (0 – 12) | 0.05 |

| 1. **Mediation modelling for cognitive decline^** | ***Mediated effect***  ***Per 1 unit increase in lncysC*** | ***p-value*** | ***% Coefficient reduction (CI)*** | ***p-value*** |
| --- | --- | --- | --- | --- |
| Univariable + Age | -0.60 (-0.72 - -0.51) | <0.001 | 72 (50 – 95) | <0.001 |
| Univariable+ Cardiovascular BS | -0.44 (-0.56- -0.31) | <0.001 | 52 (31 – 72) | <0.001 |
| Univariable +Inflammation BS | -0.01 (-0.04 – 0.02) | 0.39 | 2 (-2 – 6) | 0.40 |
|  |  |  |  |  |
| Multivariate#1*+ Age | -0.61 (-0.72 - -0.50) | <0.001 | 76 (51 – 102) | <0.001 |
| Multivariate#1+CardiovascularBS | -0.20 (-0.29 - -0.09) | <0.001 | 98 (-38 – 236) | 0.13 |
| Multivariate#1+ InflammationBS | -0.02 (-0.04 – 0.01) | 0.33 | 8 (-11 – 28) | 0.41 |
|  |  |  |  |  |
| Multivariate#2*+ Age | -0.50(-0.60 - -0.40) | <0.001 | 68 (43 – 94) | <0.001 |
| Multivariate#2+Cardiovascular BS | -0.18 (-0.28 - -0.08) | <0.001 | 75 (-13 – 163) | 0.09 |
| Multivariate#2+ Inflammation BS | -0.01 (-0.04 - 0.01) | 0.30 | 5 (-6 – 18) | 0.37 |

**Abbreviations:** BS; biomarker score, CI; confidence interval, SD; standard deviation; ln natural logarithm.

**Multivariable Model#1** was adjusted for age, and demographics (sex, education, depression).

**Multivariable Model#2** was adjusted for age, demographics (sex, education, depression), and clinical vascular risk factors (i.e., smoking, obesity, diabetes, number of CVD risk factors, systolic BP, diastolic BP, cholesterol, HDL cholesterol, CRP level).

**Multivariable Model#1*** was adjusted for demographics (sex, education, depression).

**Multivariable Model#2*** was adjusted for demographics (sex, education, depression), and clinical vascular risk factors (smoking, obesity, diabetes, number of CVD risk factors, systolic BP, diastolic BP, cholesterol, HDL cholesterol, CRP level).

^Regression models for cognitive decline were additionally adjusted for baseline cognitive score.

**Supplementary Table S5.** Mediation analysis for the association of cystatin C with memory

| ***Mediation modelling*** | ***Mediated effect***  ***per 1 unit increase in lncysC*** | ***p-value*** | ***% Coefficient reduction (CI)*** | ***p-value*** |
| --- | --- | --- | --- | --- |
| Univariable + Age | -1.21 (1.37, -1.08) | <0.001 | 64 (52 – 77) | <0.001 |
| Univariable+ Cardiovascular BS | -0.80 (-0.99- -0.60) | <0.001 | 43 (30 – 55) | <0.001 |
| Univariable+ Inflammation BS | -0.03 (-0.07, 0.01) | 0.20 | 2(-1 – 4) | 0.15 |
|  |  |  |  |  |
| Multivariable#1*+ Age | -0.99 (-1.14 - -0.84) | <0.001 | 73 (54 – 92) | <0.001 |
| Multivariable#1+ Cardiovascular BS | -0.32 (-0.06 - 0.0.1) | <0.001 | 86 (2 – 169) | 0.04 |
| Multivariable#1+ Inflammation BS | -0.02 (-0.06 – 0.01) | 0.29 | 5 (-4 – 17) | 0.30 |
|  |  |  |  |  |
| Multivariable#2*+ Age | -0.76(-0.91 - -0.62) | <0.001 | 73 (49 – 98) | <0.001 |
| Multivariable#2+ Cardiovascular BS | -0.28 (-0.42 - -0.13) | <0.001 | 90 (-2 – 220) | 0.10 |
| Multivariable#2+ Inflammation BS | -0.02 (-0.05 - 0.01) | 0.27 | 4 (-7 – 20) | 0.35 |

**Abbreviations:** BS; biomarker score, CI; confidence interval, ln; natural logarithm.

**Multivariable Model#1** was adjusted for age, and demographics (i.e., sex, education, depression).

**Multivariable Model#2** was adjusted for Multivariable Model 1, and clinical vascular risk factors (i.e., smoking, obesity, diabetes, number of CVD risk factors, systolic BP, diastolic BP, cholesterol, HDL cholesterol, CRP level).

**Multivariable Model#1******* was adjusted for demographics (i.e., sex, education, depression).

**Multivariable Model#2*** was adjusted for Multivariable Model 1* and clinical vascular risk factors (i.e., smoking, obesity, diabetes, number of CVD risk factors, systolic BP, diastolic BP, cholesterol, HDL cholesterol, CRP level).

**Supplementary Table S6.** Mediation analysis for the association of cystatin C with executive function

| *Mediation modelling* | ***Mediated effect***  ***Per 1 unit increase in lncysC*** | ***p-value*** | ***% Coefficient reduction (CI)*** | ***p-value*** |
| --- | --- | --- | --- | --- |
| Univariable + Age | -2.10 (-2.33 - -1.88) | <0.001 | 74 (62 – 85) | <0.001 |
| Univariable+ Cardiovascular BS | -1.07 (-1.30- -0.84) | <0.001 | 39 (28 – 47) | <0.001 |
| Univariable + Inflammation BS | -0.01 (-0.06 – 0.03) | 0.64 | 1 (-1 – 3) | 0.65 |
|  |  |  |  |  |
| Multivariable#1*+ Age | -1.89 (-2.10 - -1.68) | <0.001 | 81 (66 – 95) | <0.001 |
| Multivariable#1+ Cardiovascular BS | -0.32 (-0.48 - -0.16) | <0.001 | 64 (4 – 124) | 0.03 |
| Multivariable#1+ Inflammation BS | -0.02 (-0.06 – 0.02) | 0.42 | 4 (-6 – 13) | 0.44 |
|  |  |  |  |  |
| Multivariable#2*+ Age | -1.53(-1.73 - -1.34) | <0.001 | 74 (58 – 90) | <0.001 |
| Multivariable#2+ Cardiovascular BS | -0.32 (-0.48 - -0.16) | <0.001 | 53 (7 – 99) | 0.02 |
| Multivariable#2+ Inflammation BS | -0.01 (-0.06 - 0.02) | 0.41 | 3 (-4 – 9) | 0.42 |

**Abbreviations:** BS; biomarker score, CI; confidence interval, ln; natural logarithm.

**Multivariable Model#1** was adjusted for age, and demographics (i.e., sex, education, depression).

**Multivariable Model#2** was adjusted for Multivariable Model 1, and clinical vascular risk factors (i.e., smoking, obesity, diabetes, number of CVD risk factors, systolic BP, diastolic BP, cholesterol, HDL cholesterol, CRP level).

**Multivariable Model#1*** was adjusted for demographics (i.e., sex, education, depression).

**Multivariable Model#2*** was adjusted for Multivariable Model 1* and clinical vascular risk factors (i.e., smoking, obesity, diabetes, number of CVD risk factors, systolic BP, diastolic BP, cholesterol, HDL cholesterol, CRP level).

**Supplementary Table S7.** Mediation analysis for the association of cystatin C with the difference in memory between Wave 1 and Wave 3

| *Mediation modelling* | ***Mediated effect***  ***Per 1 unit increase in lncysC*** | ***p-value*** | ***% Coefficient reduction (CI)*** | ***p-value*** |
| --- | --- | --- | --- | --- |
| Univariable + Age | -0.95 (-1.09 - -1.08) | <0.001 | 70 (54 – 85) | <0.001 |
| Univariable+ Cardiovascular BS | -0.47 (-0.62- -0.31) | <0.001 | 35 (21 – 49) | <0.001 |
| Univariable + Inflammation BS | -0.00 (-0.03 – 0.04) | 0.70 | 0 (-2 – 2) | 0.70 |
|  |  |  |  |  |
| Multivariable#1*+ Age | -0.94 (-1.08 - -0.80) | <0.001 | 82 (61 – 103) | <0.001 |
| Multivariable#1+Cardiovascular BS | -0.14 (-0.25 - -0.01) | 0.02 | 85 (-87 – 257) | 0.32 |
| Multivariable#1+ Inflammation BS | 0.00 (-0.02 – 0.04) | 0.57 | -2 (-20 – 14) | 0.60 |
|  |  |  |  |  |
| Multivariable#2*+ Age | -0.79(-0.92 - -0.66) | <0.001 | 80 (56 – 105) | <0.001 |
| Multivariable#2+ Cardiovascular BS | -0.13 (-0.25 - -0.01) | 0.03 | 82 (-87 – 252) | 0.34 |
| Multivariable#2+ Inflammation BS | -0.01 (-0.03- 0.01) | 0.60 | -3 (-21 – 12) | 0.60 |

**Abbreviations:** BS; biomarker score, CI; confidence interval, ln; natural logarithm.

**Multivariable Model#1** was adjusted for age, and demographics (i.e., sex, education, depression) and baseline cognitive score in memory.

**Multivariable Model#2** was adjusted for Multivariable Model 1, and clinical vascular risk factors (i.e., smoking, obesity, diabetes, number of CVD risk factors, systolic BP, diastolic BP, cholesterol, HDL cholesterol, CRP level) and baseline cognitive score in memory.

**Multivariable Model#1*** was adjusted for demographics (i.e., sex, education, depression) and baseline cognitive score in memory.

**Multivariable Model#2*** was adjusted for Multivariable Model 1* and clinical vascular risk factors (i.e., smoking, obesity, diabetes, number of CVD risk factors, systolic BP, diastolic BP, cholesterol, HDL cholesterol, CRP level) and baseline cognitive score in memory.

**Supplementary Table S8.** Mediation analysis for the association of cystatin C with the difference in executive function between Wave 1 and Wave 3

| *Mediation modelling* | ***Mediated effect***  ***Per 1 unit increase in lncysC*** | ***p-value*** | ***% Coefficient reduction (CI)*** | ***p-value*** |
| --- | --- | --- | --- | --- |
| Univariable+ Age | -0.89 (-1.02 - -0.76) | <0.001 | 55 (44 – 66) | <0.001 |
| Univariable+ Cardiovascular BS | -0.50 (-0.62- -0.34) | <0.001 | 31 (21 – 41) | <0.001 |
| Univariable + Inflammation BS | -0.00 (-0.03 – 0.03) | 0.85 | 0 (-2 – 2) | 0.85 |
|  |  |  |  |  |
| Multivariable#1*+ Age | -0.90 (-1.03 - -0.77) | <0.001 | 57 (45 – 68) | <0.001 |
| Multivariable#1+ Cardiovascular BS | -0.19 (-0.28 - -0.07) | <0.001 | 28 (7 – 48) | <0.001 |
| Multivariable#1+ Inflammation BS | -0.01 (-0.04 – 0.03) | 0.57 | 1 (-4 – 5) | 0.58 |
|  |  |  |  |  |
| Multivariable#2*+ Age | -0.76(-0.89 - -0.64) | <0.001 | 52 (40 – 63) | <0.001 |
| Multivariable#2+ Cardiovascular BS | -0.16 (-0.26 - -0.06) | 0.003 | 24 (5 – 43) | 0.01 |
| Multivariable#2+ Inflammation BS | -0.00 (-0.03 - 0.02) | 0.52 | 1 (-3 – 5) | 0.53 |

**Abbreviations:** BS; biomarker score, CI; confidence interval, ln; natural logarithm.

**Multivariable Model#1** was adjusted for age, and demographics (i.e., sex, education, depression) and baseline cognitive score in executive function.

**Multivariable Model#2** was adjusted for Multivariable Model 1, and clinical vascular risk factors (i.e., smoking, obesity, diabetes, number of CVD risk factors, systolic BP, diastolic BP, cholesterol, HDL cholesterol, CRP level) and baseline cognitive score in executive function.

**Multivariable Model#1*** was adjusted for demographics (i.e., sex, education, depression) and baseline cognitive score in executive function.

**Multivariable Model#2*** was adjusted for Multivariable Model 1* and clinical vascular risk factors (i.e., smoking, obesity, diabetes, number of CVD risk factors, systolic BP, diastolic BP, cholesterol, HDL cholesterol, CRP level) and baseline cognitive score in executive function.
